# Human Resource Management and Innovative Performance in Non-profit Hospitals: The Mediating Effect of Organizational Culture

**DOI:** 10.3389/fpsyg.2020.01422

**Published:** 2020-06-19

**Authors:** Julio C. Acosta-Prado, Oscar H. López-Montoya, Carlos Sanchís-Pedregosa, Rodrigo A. Zárate-Torres

**Affiliations:** ^1^School of Business Sciences, Universidad del Pacífico, Lima, Peru; ^2^School of Accounting, Economic and Business Sciences, Universidad de Manizales, Manizales, Colombia; ^3^School of Administration, Universidad del Tolima, Ibagué, Colombia; ^4^School of Economic and Business Sciences, Universidad de Sevilla, Seville, Spain; ^5^Colegio de Estudios Superiores de Administración, Bogotá, Colombia

**Keywords:** human resource management, innovative performance, organizational culture, PLS-SEM, non-profit hospitals

## Abstract

Literature suggests that human resources of non-profit hospitals (NPHs) present features that could potentially reach any expected organizational performance even when the attention to human resource management (HRM) are often low in non-profit organizations. Nowadays ambitious organizations strive to obtain a profitable performance that is also innovate and do it through building an organizational culture (OC), while for NPHs a positive culture is given by their human resources traits. However, there is not enough literature to understand how these three variables behave together. This study aims to explain the influence of HRM on IP mediated by OC. The research model was assessed through Partial Least Squares Structural Equation Modeling (PLS-SEM). The results support all the stated hypotheses. Both, HRM and OC are moderately strong predictors of IP, and OC mediates partially and in a complementary way the relationship between HRM on IP. An importance-performance map analysis (IPMA) was performed to expand the PLS-SEM results. The OC indicators show greater importance to explain IP, consequently, they are the most relevant indicators to initiate management actions by NPHs. The influence of HRM on IP represent an opportunity for NPH as it implies an affordable investment in comparison to the cost of technological solutions for enterprises.

## Introduction

Non-profit hospital (NPH) is a governmental or private organization that orient their services to low-income population. For this reason, NPH’s patients are not expected to have the money to pay for advanced medical care, however, medical advancements have a great deal with providing effective treatment and covering a wider scope of illnesses than regular medicine does. For this reason, the use of innovation in NPHs marks the quality of the health services they provide.

Current organizations follow a vision that involves innovation as it guarantees their success and survival in an evolutionary environment and in outcome terms it is translated as an innovative performance (IP). Innovation is needed to redefine business models in order to make them more affordable for low- and middle-income people. In developing countries, the organizational IP has redesigned cost structure that make products and services more affordable and accessible (George et al., 2015). For this reason, social innovation represents an opportunity to democratize access to basic medical services (Michelini, 2012; Christensen et al., 2015) through the implementation of innovative solution in non-profit hospitals. Actually, non-profit organizations orient their IP to social innovation that leads to aims more aligned to their users’ expectation (Westley et al., 2014; Phillips et al., 2015).

Particularly, NPH’s hold a responsibility to be innovative when delivering medical care. An IP for this organizations consists on answering the diverse needs of patients by new projects, processes or services (Sanzo-Perez et al., 2015; Jaskyte, 2018, 2020; Brimhall, 2019). In other words, innovation comes from the introduction of new ideas or practices that leads to the improvement of the treatment, the diagnose, prevention, research or education (Omachonu and Einspruch, 2010). Moreover, social innovation involves collaborations to implement solutions to social problems, particularly at local level (Brandsen et al., 2016). Solutions are assumed to have positive societal effects, either through increasing aggregate utilitarian value, or by empowering citizens in innovation processes (Ayob et al., 2016).

In this scenery, NPHs limit their performance to a tight budget and therefore have less chances to innovate. There are less resources and yet a constant need to adjust to a competitive environment (Svensson et al., 2019). In particular, aside the financial resources, human resources are other highly relevant resources to pursue an IP. Indeed, the impact of human resource management (HRM) reaches individual achievements regarding skills and motivation amongst other personal traits that are pre-requisites for innovation (Diaz-Fernandez et al., 2017). That is to say, IP, understood as the capacity of a business to obtain new products and other outputs, is strongly related to HRM (Adnan et al., 2016; Métailler, 2016; Diaz-Fernandez et al., 2017). Through the activities undertaken by HRM, namely building a culture that values new ideas or enabling employees to keep growing professionally, it is noticeable how they influence on the employees and consequently impact on IP as well (Bal et al., 2014; Liao and Huang, 2016; Diaz-Fernandez et al., 2017; Gile et al., 2018; Jaskyte, 2018; Meyer and Leitner, 2018).

The implementation of HRM’s strategies shapes the organizational culture (OC). In particular, an innovative culture might lead to an IP. Mesch (2010) pointed out that HRM is the main element to reach an effective organizational performance in non-profit organizations as in other organization types. For this reason, HRM are likely to influence OC within the particular context of NPH (Jaskyte, 2011, 2015, 2018; Brown et al., 2016; Aktar and Pangil, 2017; Baluch, 2017). Furthermore, OC is recognized by its role to dynamize the IP (Peters and Waterman, 1982; Fondas and Denison, 1991; Ahmed, 1998; Jaskyte and de Riobó, 2004; Jaskyte, 2015; Meyer and Leitner, 2018). This relationship has been also studied in contexts such as NPH finding significant results (McDonald, 2007; Brimhall, 2019; Narapareddy and Berte, 2019). Hence, OC is likely to influence IP directly and mediate the influence of HRM on IP too.

NPHs face difficulties to reach the expected health service, they do not often count with the technology needed, the specialties, or the medicine, to mention some of their many limitations. Nevertheless, they are the best affordable option for most of the population. Due to that fact, it is necessary they have alternative strategies to rise their service quality through innovation. Bearing that in mind, this study aims to examine the relationship between HRM and IP, while considering the mediating effect of OC. In order to do so, the research question is stated as follows: What is the direct effect of HRM on IP, mediated by OC, in NPH?

This study is divided into five sections. The first section has introduced the topic and elaborated on the research problem this study aims to address. Section “Literature Review” presents the theoretical framework regarding this study’s main constructs. The procedure followed to obtain and analyses the data is presented “Materials and Methods,” while the results are explained in section “Results and Discussion.” Finally, section “Conclusion and Recommendations for Future Studies” discusses the findings of this study and give directions for future research.

## Literature Review

This section elaborates on the constructs involved in this study’s theoretical model. It addresses the theory reviewed about the management of the human resources, the updated literature on innovation regarding the organization performance, and finally, the organizational culture’s literature is pinpointed as a mediator among the other two constructs.

### Human Resources Management

Human resources management holds a strategic role to reassure organizational effectiveness through the human resources of a company. In fact, according to the resources based theory, HRM is responsible of managing part of the strategic resources of the organization to enable firms’ growth and competitive advantages for a superior performance (Wernerfelt, 1984; Barney, 1991; Conner, 1991; Grant, 1991; Collis and Montgomery, 1995; Teece et al., 1997). The Resource-Based theory analyzes and interprets the strategic internal resources of organizations such as: resources, capacities, organizational processes, information, knowledge, among others that enable to develop and maintain competitive advantages (Barney, 1991).

However, the globalized market requires companies a strategic envision of their knowledge resource. Kang et al. (2007) elaborates on this theory and came up with the knowledge-based theory that give human resources a relevant meaning in the process of value creation. Doing so, this theory links organizational learning, social relations and HRM into the same knowledge flow. That is to say, HRM is crucial at fostering innovation processes in companies (Li et al., 2006) by influencing creativity (Jiang et al., 2012) and knowledge management system (Jiménez-Jiménez and Sanz-Valle, 2011). Moreover, knowledge-based perspective regarding organizational capacities outreached by HRM are related to OC and have an impact on innovation success (Long and Fahey, 2000; Leal-Rodríguez et al., 2014).

In the particular contexts of non-profit organizations, prior research stated that even when the investment on HRM is less than the standard, it still earns positive outcomes (Nickson et al., 2008; Baines, 2010; Atkinson and Lucas, 2013; Ariza-Montes and Lucia-Casademunt, 2016; Baluch, 2017). Yet strategies on HRM have demonstrated to bring good results in employees that later on are beneficial to the organization’s performance as earlier stated. A key factor to success of non-profit organizations is the ability to identify and develop capacities through their available resources. For this reason, resources that has been proven to be useful in past experiences such as culture, HRM, among others, are considered strategic (Colbert, 2004; Akingbola, 2013; Oliveira and Toda, 2013; Brown et al., 2016; Gile et al., 2018).

### Innovative Performance

Nowadays, innovative organizations have more chances to survive in the competitive global market. Innovation is a complex term and most of the time is related to technology, however, is not only limited by the scientific and technological dimensions (Echevarría, 2008). A general approach states that “innovation is the implementation of a new or significantly improved product (good or service), or process, a new marketing method, or a new organizational method in business practices, workplace organization or external relations” (OECD/Eurostat, 2005). To complement this approach, Gurrutxaga (2011) disaggregates innovation in the ability to create and innovate as options to respond to social necessities, defining structural constraints and rescuing the relevance of innovation.

Regarding the healthcare sector, innovation is the introduction of a new concept, idea, service, process or product to improve treatment, diagnosis, education, prevention and research, aiming a long-term objective to improve quality, safety, results, efficiency and costs (Omachonu and Einspruch, 2010). This means that novel practices on the benefit of effective healthcare favors to reduce rates of mortality and morbidity. Based on the patients’ perspective, the benefit of healthcare sector innovation is evident through improved health or reduced suffering due to illness (Faulkner and Kent, 2001). In other words, innovation has a wide scope to be implemented in this sort of services.

It is necessary to notice that mostly all definitions regarding innovation come from a for-profit organization approach regardless the nature of other types of organizations such as non-profit ones. However, non-profit organizations outreach successful levels of innovation that are aligned to social innovation initiatives. Svensson et al. (2019) consider that innovation in non-profit organizations, social innovation, happens with “better ways of achieving meaningful impact in addressing a given social issue and promoting positive social change.” This put non-profit organizations in a double pressure situation to keep financially stable and maintain social performance toward meeting their mission and satisfying numerous stakeholders (Landrum, 2007). Overall, NPHs aiming to perform innovatively address healthcare in a way that makes a social change which means a social innovative performance capable to reach not just a change, but a shareable social value creation (Hwang and Christensen, 2008; Michelini, 2012) that will end up in a more inclusive healthcare service and social nosiness models (Vecchio and Rappini, 2011; Michelini, 2012; Angeli and Jaiswal, 2016).

According to the above, IP is the consequence of a set of variables, however, HRM and OC are relevant when it comes about particular organizations such as NPHs. For this reason, this study seeks to explain the influence of HRM on IP and OC on IP. These objectives determine the following hypotheses:

H1: HRM influences positively the IP.
H3: OC influence positively the IP.

### Organizational Culture

The organizational culture is defined as the set of intergroup interrelationships that manifests mainly when changing opportunities and threats take place in the organization’s environment. In the healthcare sector, adaptation to new situations could determine life or death which remains mandatory for them to build an organizational culture that could handle this environment. Nevertheless, NPHs face a challenge on this matter as their resources and vision limit their possibilities to act upon an organizational culture strategy.

Many studies has determine the influence of HRM on OC and how highly related are both variables (Hartog et al., 2004; Kusluvan et al., 2010; Aktar and Pangil, 2017). It means the implementation of strategies to build and environment where human resources are capable to impact on the innovative outcomes by the right knowledge acquisition, distribution and storage by employees. Ballesteros-Rodríguez et al. (2012) point that culture define how things are done and influence leaders to stablish objectives and practices for HRM. For this reason, HRM and OC seems to have a relationship that works in a bidirectional way.

OC plays a leading role in achieving to reach the expected IP (Jaskyte and Dressler, 2005; Jaskyte, 2018). Furthermore, the literature recognizes the role of principles and climate of the organization as elements of the OC that enable an IP (Peters and Waterman, 1982; Fondas and Denison, 1991; Ahmed, 1998; Jaskyte, 2004, 2015; Meyer and Leitner, 2018). This approach has also been developed regarding the health sector (McDonald, 2007; Brimhall, 2019; Narapareddy and Berte, 2019) but not the non-profit hospitals in particular. For this reason, OC influence IP while it is affected by HRM, then HRM influence on IP might be influenced by the mediator role of OC.

The proposed model is theoretically based on mediation because a mediating variable (OC) intervenes between two constructs that are related (HRM and IP). This implies that a change in HRM will lead to a change in OC, which in turn will generate a change in the endogenous variable IP. What this mediation model seeks is to analyze the intensity of OC relationships with the other constructs, justifying the mechanisms underlying the cause-effect relationship between HRM and IP. Unlike what a moderation model seeks, where the intensity or sense of the relationship between variables depends on a third variable that does not directly interact with the exogenous or endogenous variable (Nitzl et al., 2016).

In line with the above, this study analyses how HRM influence OC. Besides it is assessed the mediator role of OC in the relationship between HRM and IP. These objectives are stated in the following hypotheses:

H2: HRM influences positively the OC.
H4: OC mediates the relationship between HRM and IP.

**FIGURE 1:**
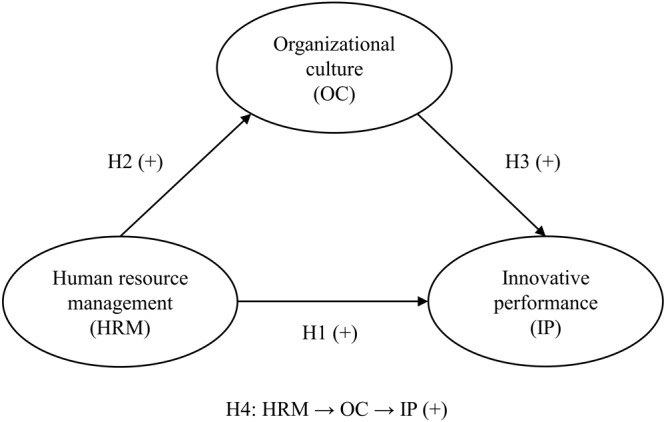
Theoretical model and research hypotheses; HRM, human resource management; OC, organizational culture; IP, innovative performance.

Figure 1 represents graphically the theoretical model for this study and the hypotheses associated to it.

## Materials and Methods

### Design

According to the research designs classification system in Psychology by Ato et al. (2013) this study belongs to an empirical study. The strategy was associative as it explores the functional relationship among the three variables under analysis. The type of study is explanatory, where a mediation model was tested. Finally, latent variables design (LVD) or structural equation modeling (SEM) was used, consisting of two parts that make up the model: one structural or inner model (the relationship among the constructs) and the other measurement or outer model (the relationship between the indicators and the constructs they measure). The statistical procedure to estimate the parameters of the SEM model was based on the variances, also known as Partial Least Squares (PLS-SEM).

### Participants

The study sample was made up of health sector personnel working in Colombian non-profit hospitals (NPHs). These are public, private or mixed organizations that provide medical care in three levels: (1) care by general, technical and auxiliary personnel, with low complexity technology, (2) care by specialized personnel, with medium complexity technology, and (3) care by specialized personnel and subspecialized, with the technology of the highest complexity (Prada-Ríos et al., 2017). Only third level NPHs participated and were made up of regional, university and specialized hospitals.

**FIGURE 2:**
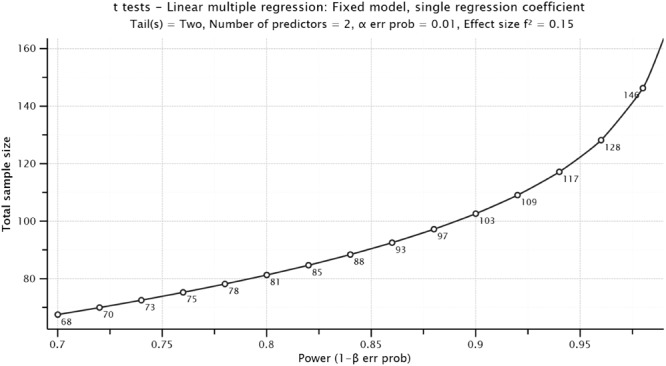
Minimum recommended sample size based on *a priori* power analysis.

Participants were selected through non-probability, intentional sampling (Kerlinger and Lee, 2000). *A priori* statistical power analysis was computed to obtain the minimum sample size. This minimum sample size ensures that the results of the statistical method are robust and that the model is generalizable. The analysis was performed using the G^∗^Power 3.1.9.7 software (Faul et al., 2009). This statistical procedure requires to previously set input parameters, namely a significance level of 0.01 (two tails); an expected statistical power of 0.95, higher than the recommended by Cohen (1992) expected effect size (*f*^2^) of 0.15 or moderate effect; and two predictors. After the analysis, the minimum recommended sample size was determined to be 123 (Figure 2).

The sample size was made up of 162 Colombian NPHs workers, however, only 150 were finally included in the study. 12 measurement scales were rejected as they presented more than 15% of incomplete answers and three items or more unanswered (Sarstedt and Mooi, 2019). This resulted in a 92.59% valid response rate. Based on the 150 participants, the time they have been working in the NPHs ranged from 1 to 30 years (*M* = 6.046, *SD* = 5.72), 65.3% were women, 31.3% were doctors, almost all (99.3%) belonged to public NPHs, and 34.7% had undergraduate studies. Table 1 presents the complete sociodemographic characteristics of the participants.

**TABLE 1:** Sociodemographic characteristics of the participants (*n* = 150).

**Characteristic**	***n***	***%***
Gender		
Female	98	65.333
Male	44	29.333
Missing	8	5.333
Entailment		
Auxiliaries	35	23.333
Manager or administrative staff	30	20.000
Nurses	33	22.000
Doctors	47	31.333
Missing	5	3.333
Type		
Private	1	0.667
Public	149	99.333
Academic degree		
Doctoral	3	2.000
Master	10	6.667
Specialization	50	33.333
Undergraduate	52	34.667
Technician	29	19.333
No academic degree	1	0.667
Missing	5	3.333

### Instrument

All the study variables were measured through a measurement scale with satisfactory psychometric properties developed by López et al. (2018a). It measures a set of variables related to innovation in the health sector. For this study, three of these variables were considered: human resources management (measured through 10 items, from HRM–1 to HRM–10), organizational culture (measured through two items, OC–1 and OC–2) and innovative performance (measured through three items, from IP–1 to IP–3). The items were five points Likert scales, ranging from 1 (no activity has been carried out to improve the characteristic of interest) to 5 (positive results have been obtained against the aspect investigated).

The instrument was developed in a sample of 107 Colombian hospitals workers between 2016 and 2017, and the variables associated with innovation in health organizations were selected from a systematic review. In the original scale, the three variables had high levels of internal consistency measured through the alpha coefficient (López et al., 2018b). Besides, it has validity evidence based on the internal structure, through exploratory and confirmatory factor analysis (López et al., 2018b). This sequence of analyses indicate that the instrument meets the psychometric properties required for a measurement scale in the social and health sciences (Acosta-Prado et al., 2020).

### Procedure

To collect the data, a form in Google Forms was sent via email to all members of the NPHs. The instrument included information regarding the research objectives. Before sending the email, all workers gave their consent to participate in the study. The responses were stored into a spreadsheet on Google Drive, while taking care of the anonymity of the participant responses. The research was carried out following the Declaration of Helsinki.

The missing data, that is to say not-answered items, were eliminated when they reached more than 15% of unanswered items per case. The remaining missing data was replaced with the mean of the valid values of the correspondent indicator. This method was used because its ease to use and the missing values were found less than 5% of the values per indicator (Hair et al., 2017).

### Data Analysis

For the statistical analysis, the Partial Least Squares (PLS), an approximation of the variance-based structural equation modeling (SEM) was used. This technique was selected due to the properties of the constructs that are part of the research model (Ali et al., 2018). PLS-SEM estimates coefficients that maximize the explained variance of endogenous constructs, giving the predictive characteristic to this statistical technique (Mateos-Aparicio, 2011). SmartPLS 3.2.9 software (Ringle et al., 2015) was used for this study.

Among the advantages of the PLS-SEM (Hair et al., 2011) are (a) the absence of identification problems with reduced sample sizes, usually reaching high levels of statistical power with small sample sizes; (b) the data distribution does not involve assumptions as it is a nonparametric method; (c) its robustness in the presence of missing values, as long as they are below a reasonable limit; (d) the amount of unexplained variance is reduced; and (e) the reliability and validity of the measurement models are assessed using various criteria.

In PLS-SEM, the structural model describes the relationships among the latent variables (constructs) and the measurement model represent the relationships between the constructs and their corresponding indicators. Regarding the latter, two specification categories must be considered: reflective and formative measurement models (Rigdon, 2012). In reflective models, the indicators are the effects or manifestations of an underlying construct; on the contrary, in formative models, the indicators form the construct using linear combinations (Jarvis et al., 2003). In this study, a reflective measurement model was used, where reflective indicators support the idea that the construct causes the measurement or covariation of the indicators. Likewise, in the structural model, HRM is an exogenous latent variable, whereas OC and IP are endogenous latent variables.

Before the PLS-SEM analysis, it was verified that the data do not deviate excessively from a normal distribution since data that are far from normal are problematic at evaluating the significance of the parameters. Specifically, these data overestimate the standard errors obtained by bootstrapping which reduces the possibility to consider some path coefficients as statistically significant (Henseler et al., 2009). Therefore, the kurtosis and skewness of the data distributions (indicators) were examined, and values between −1 and +1 were considered appropriate (Hair et al., 2019a). Besides, the mean was estimated as a measure of central tendency and the standard deviation as a measure of variability, both descriptive statistics give an idea of how the participants responded to the items on the measurement scale.

The PLS-SEM results are analyzed following a systematic process. The evaluation of the quality of the measurement and structural models focuses on statistics that indicate the predictive capacity of the model (Hair et al., 2013). Regarding the reflective measurement model, the reliability was assessed by the internal consistency method, using the alpha, rho_A, and composite reliability (CR) coefficients, as well as the reliability of the individual indicator and the average variance extracted (AVE) to evaluate convergent validity. The assessment of the reflective measurement model also included discriminant validity. The Fornell and Larcker criteria and the heterotrait-monotrait ratio (HTMT) were used to examine the discriminant validity. In the structural model, the measures considered were *R*^2^ (explained variance), *f*^2^ (effect size), *Q*^2^_predict_ (predictive performance), and the magnitude and statistical significance of the path coefficients.

To test the statistical significance of the coefficients, the non-parametric bootstrap procedure was used with 10,000 bootstrap samples without sign change (Streukens and Leroi-Werelds, 2016). The bootstrap process provides the standard error for any estimated coefficient and this error serves as the basis to determine the empirical value of *t* and its associated *p*-value. Likewise, the bias-corrected and accelerated (BCa) bootstrap-based method was used to construct the confidence intervals at a 95% confidence level and two tails (Henseler et al., 2009).

Finally, an importance-performance map analysis (IPMA) was performed to expand the PLS-SEM results, adding a dimension to the analysis that considers the scores’ mean values of the latent and observable variables (Höck et al., 2010). The objective of this analysis was to identify the antecedent constructs and indicators that have relatively high importance in the objective construct (innovative performance) but at the same time relatively low performance. The latter is important to detect potential areas that should receive more attention (Nigel, 1994).

## Results and Discussion

### Descriptive Analysis of the Items

Both skewness and kurtosis are close to zero for all the items (Table 2), indicating that the response pattern corresponds to a normal distribution. Specifically, the kurtosis values varied between −0.778 (HRM–4) and 0.223 (OC–1), while the skewness coefficients fluctuated between −0.723 (OC–1) and 0.057 (HRM–8), that are below the limits indicated by Hair et al. (2019b). Likewise, the mean and standard deviation had similar values throughout all the items. The mean was close to 3 and the standard deviation was around 1 (Table 3).

**TABLE 2:** Measurement scale to human resource management, organizational culture, and innovative performance.

**Code**	**Indicator**
HRM–1	Develop competencies with the purpose of increasing the performance of collaborators.
HRM–2	Promote the development of teamwork skills.
HRM–3	Create opportunities for professional growth for employees at the hospitals.
HRM–4	Promote the rise of those employees who meet the established goals.
HRM–5	Allow collaborators freedom so that they can make decisions regarding their work activities.
HRM–6	Keep in the clinic or hospital those people with excellent job performance.
HRM–7	Make sure that a clinic or hospital is focused on the development of people.
HRM–8	Evaluate novel ideas by collaborators.
HRM–9	Promote an environment that encourages the generation of new ideas among its collaborators.
HRM–10	Promote collaboration between members of the organization.
OC–1	The values of the organization are the permanent guide in the innovation processes.
OC–2	Create a work environment that fosters innovation processes.
IP–1	Incursion with new services to its users.
IP–2	Permanently develop innovative projects.
IP–3	Generate new processes in the hospitals (new ways of doing everyday work, new surgical procedures, new systems).

**TABLE 3:** Descriptive statistics and measurement model.

**Variable**	***M***	***SD***	***Ku***	***Sk***	**Outer loadings**	**Weights**
**Human resource management**						
HRM–1	3.300	1.044	−0.254	−0.238	0.861	0.131
HRM–2	3.400	1.033	−0.161	−0.352	0.868	0.127
HRM–3	3.173	1.088	−0.483	−0.194	0.857	0.117
HRM–4	2.907	1.151	−0.778	−0.054	0.812	0.110
HRM–5	3.200	1.172	−0.770	−0.296	0.763	0.102
HRM–6	3.320	1.127	−0.464	−0.460	0.774	0.106
HRM–7	3.167	1.003	−0.472	0.019	0.857	0.113
HRM–8	3.033	1.092	−0.576	0.057	0.817	0.113
HRM–9	3.013	0.993	−0.478	−0.109	0.903	0.140
HRM–10	3.312	0.984	−0.025	−0.293	0.857	0.130
**Organizational culture**						
OC–1	3.620	1.024	0.223	−0.723	0.938	0.530
OC–2	3.302	1.073	−0.230	−0.464	0.939	0.536
**Innovative performance**						
IP–1	3.413	1.053	−0.525	−0.305	0.910	0.363
IP–2	3.167	1.104	−0.457	−0.336	0.933	0.399
IP–3	3.233	1.048	−0.356	−0.271	0.899	0.331

*M, mean; SD, standard deviation; Ku, kurtosis; Sk, skewness.*

### Measurement Model Evaluation

Internal consistency reliability was the first criterion to evaluate. This indicates the degree of consistency among indicators to measure the constructs. The alpha coefficient, rho_A, and CR were examined and above the critical threshold of 0.700 (Nunnally and Bernstein, 1994). The HRM variable was the one that obtained the highest values in the three coefficients. Due to these results, it can be concluded that the scores obtained by the participants of the study sample in the three constructs presented adequate levels of reliability.

The convergent validity refers to the degree of positive correlation between one measure and other alternative measures of the same construct. For this purpose, the size of the outer loadings, commonly called indicator reliability, was analyzed. All the indicators of the reflective constructs presented loadings equal to or greater than 0.763 (HRM–5), above the criterion of 0.708 (Fornell and Larcker, 1981) see Table 3. Also, the AVE was calculated and it represents the mean value of the commonality of the indicators of certain construct. The three constructs presented values greater than 0.700 (Table 4), showing a very good level of convergent validity (Moral, 2019).

**TABLE 4:** Correlation matrix, reliability, convergent and discriminant validity, and heterotrait-monotrait ratio (HTMT).

**Variable**	**Alpha**	**rho_A**	**CR**	**AVE**	**HRM**	**OC**	**IP**
Human resource management	0.953	0.957	0.959	0.702	0.838*		
Organizational culture	0.864	0.864	0.936	0.880	0.737	0.938*	
Innovative performance	0.902	0.910	0.938	0.836	0.684	0.717	0.914*
**Heterotrait-monotrait ratio (HTMT)**							
Human resource management					0.673^†^		
Organizational culture					0.808	0.763^†^	
Innovative performance					0.730	0.808	0.754^†^

*Square root of the AVE; ^†^average inter-item correlations; alpha, alpha coefficient; rho_A, coefficient rho_A; CR, composite reliability; AVE, average variance extracted; HRM, human resource management; OC, organizational culture; IP, innovative performance.

Concerning discriminant validity, it informs to what extent a construct is different from other constructs. The first method used was the Fornell and Larcker (1981) criterion, which compares the square root of the AVE values with the correlations of the latent variables, where the first must be greater. As a second method, the HTMT ratio of the correlations was used because it is the best criterion in PLS-SEM to assess discriminant validity, and it requires, as appropriate values, numbers under 0.85 (Henseler et al., 2015). The results of the study indicate that both the Fornell and Lacker criteria and the HTMT ratio obtained satisfactory levels (Table 4). Furthermore, the HTMT for HRM and OC was 0.808 [0.702; 0.895], for HRM and IP it was 0.730 [0.612; 0.823], and for OC and IP it was 0.808 [0.694; 0.889]. The HTMT confidence interval did not include 1 (Franke and Sarstedt, 2019).

### Structural Model Evaluation

**FIGURE 3:**
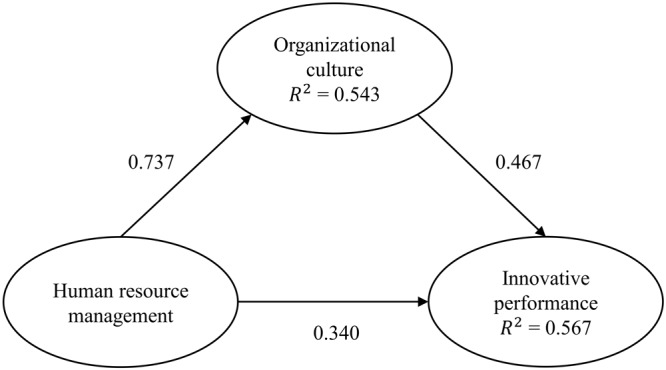
Assessment results of path coefficients and variance explained (*R*^2^).

The results of the structural model (Table 5) show that OC has a direct effect on IP (0.467), followed by HRM (0.340). Likewise, the two constructs explain 56.7% of the variance of the endogenous construct IP (*R*^2^ = 0.567), as observed in Figure 3. HRM also explains 54.3% of the variance of OC. On the other hand, based on the magnitudes of the path coefficients, all the relationships were statistically significant (Table 5). Regarding effect sizes, Cohen (1988) assessment was performed with values of *f*^2^ > 0.02, *f*^2^ > 0.15, and *f*^2^ > 0.35 that represent small, medium, and large effect sizes, respectively. Results indicate a small effect on H1, a medium effect on H3, and a large effect on H2. In summary, both HRM and OC are moderately strong predictors of IP (Table 5).

**TABLE 5:** Structural model evaluation.

**Hypotheses**	**Path coefficient**	***t*-Statistic**	**95% BCa**	***f^2^***	***R*^2^**	***Q*^2^_predict_**
H1: HRM → IP	0.340	3.852***	[0.156; 0.496]	0.122	0.567	0.459
H2: HRM → OC	0.737	16.238***	[0.628; 0.808]	1.188	0.543	0.537
H3: OC → IP	0.467	5.251***	[0.275; 0.632]	0.230		
H4: HRM → OC → IP	0.344	5.048***	[0.207; 0.484]			

***p < 0.001.

In addition, the statistical significance (*t* = 5.048, *p* < 0.001) of the indirect effect of HRM on IP through OC (0.344) was observed. From these results, a complementary mediation is given, where both the indirect effect and the direct effects are statistically significant and point in the same direction (Nitzl et al., 2016). That is to say, OC mediates partially and in a complementary way the relationship between HRM on IP.

Finally, the *Q*^2^_predict_ indicator was examined to assess the predictive performance of the structural model. If the *Q*^2^_predict_ value is positive, the prediction error of the PLS-SEM results is less than the prediction error of simply using the mean values. *Q*^2^_predict_ values were interpreted with Hair et al. (2019a) rule of thumb and values of 0.01, 0.25, and 0.50, respectively, show small, medium and high-relevance situations of a model. Besides, a medium relevance for H1 and great relevance for H2 were found (Table 5).

### Importance-Performance Map Analysis

In this analysis, the total effects represent the importance of the background indicators and constructs to explain the objective variable (innovative performance), while the mean scores of the variables represent their respective achieved performances (Höck et al., 2010). Results show that HRM has a performance of 55 and OC of 62 (Table 6). The constructs show relatively high performance. HRM expresses greater importance at predicting innovative performance (Table 6).

**TABLE 6:** Summary of importance-performance map analysis (IPMA) data.

**Variable**	**Importance**	**Performance**
Human resource management	0.684	54.680
HRM–1	0.090	57.500
HRM–2	0.087	60.000
HRM–3	0.080	54.333
HRM–4	0.075	47.667
HRM–5	0.070	55.000
HRM–6	0.073	58.000
HRM–7	0.077	54.167
HRM–8	0.077	50.833
HRM–9	0.096	50.333
HRM–10	0.089	57.790
Organizational culture	0.467	61.589
OC–1	0.247	65.500
OC–2	0.250	57.550

**FIGURE 4:**
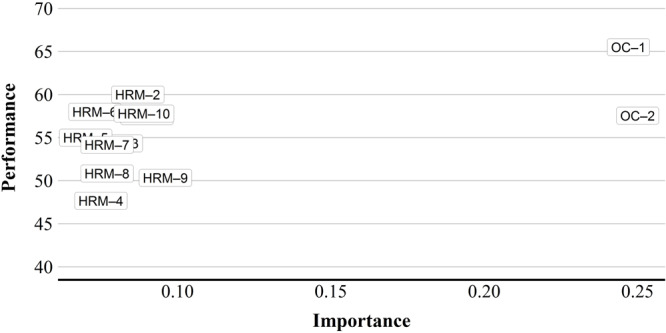
Importance-Performance map (indicators).

In Figure 4, the IPMA is presented at the level of indicators seeking to identify relevant and specific areas to improve. In this way, the weights are interpreted as the relative importance of one indicator compared to the other indicators in the measurement model. HRM indicators present a high performance but low importance for IP, so they have a lower priority when looking for improvements in their performance. On the contrary, the OC indicators show greater importance to explain innovation performance, consequently, they are the most relevant indicators to initiate management actions by NPHs.

## Conclusion and Recommendations for Future Studies

Based on the analysis of the data, all the hypotheses are supported. Specifically, the influence of HRM over IP is statistically significant. Meanwhile, OC partially mediates this relationship in hospitals working in a non-profit basis. According to this evidence, IP is explained by the strategy to manage the human resources and by the underpinning culture in this type of hospitals.

These findings are evidence to support the knowledge-based theory which means that the theory is also applicable to NPH’s context. As stated by Kang et al. (2007) HRM plays an important role in the value creation process that, in the case of NPH, bears new practices such as process, service or product to improve efficacy of results. Besides, the significance of these relationships is aligned with prior studies that also pointed out the impact of HRM on IP (Adnan et al., 2016; Métailler, 2016; Diaz-Fernandez et al., 2017). Similarly, the relationship among both variables was previously studied and found to be at the other direction which may indicate that both are linked in a cyclic pattern where HRM influence IP and vice versa. Furthermore, this result entitles a chance for NPH’s to pursue innovation and at the same time reach a competitive advantage in the market (Wernerfelt, 1984; Conner, 1991; Grant, 1991; Collis and Montgomery, 1995; Teece et al., 1997).

IP has shown to be significantly influenced by OC according to this study’s results which is also aligned with prior research (Jaskyte, 2018, 2015). Innovation success has been previously associated with OC and organizational innovation capabilities (Long and Fahey, 2000; Leal-Rodríguez et al., 2014). There is evidence that organizational principles and climate are components of organizational culture and have more implications on the innovative processes (Peters and Waterman, 1982; Fondas and Denison, 1991; Ahmed, 1998; Jaskyte, 2004, 2015; Meyer and Leitner, 2018). However, this study did not explore these further dimensions remaining the study of OC as a composite construct for future research.

This study assessed the role of OC as a mediator variable between HRM and IP. This relationship still considered a direct link between these variables, so OC played a partial mediation. The data was actually consistent with these theoretical relationships stated in the first place. That is to say, even the nature of organizations for prior findings regarding this topic, namely private companies, OC behaves similarly in non-profit hospitals. This also supports the positive influence of OC over IP found in non-profit organizations (Jaskyte, 2004, 2011, 2018; Bal et al., 2014; Langer and LeRoux, 2017; Shier et al., 2019). Thus, NPHs outreach a social innovation performance that promotes a more inclusive healthcare and depict good example for other kind of social business models (Vecchio and Rappini, 2011; Michelini, 2012; Angeli and Jaiswal, 2016).

This study found support for the stated hypotheses, however, it also faced some limitations. Regarding the sample group, of the 150 participants, only one is part of a private non-profit hospital which limit the possibility to extent these results to them. Regarding the OC variable, there was not chance to include organizational principles and climate as dimensions to assess whether they influence the most to IP as found for profit organizations. Regarding the methodological limitations of the study, all the variables were measured through a self-report instrument, whose responses could be influenced by personal biases of the workers, especially in variables such as innovative performance, where it is mainly based on the perception of same. Therefore, future studies are needed to clarify these findings.

A favorable methodological aspect of the study was the use of PLS-SEM. Among the advantages of using PLS-SEM is that it allows researchers to estimate complex models with various constructs and indicators, without requiring distributional assumptions of the data (Hair et al., 2017). These methodological advantages are supported by the PLS-SEM own conceptualization, which has a causal-predictive approach (Hair et al., 2019b). PLS-SEM should be considered not only as a less demanding measure than CB-SEM but as a complementary approach in the SEM context. Thus, PLS-SEM can be used in a wide variety of research environments, obtaining high efficiency in parameter estimation, reflected in a greater statistical power of the analysis (Hair et al., 2013).

However, this technique also has some limitations, mainly when seeking to test or confirm theoretical models, because PLS-SEM does not have fully established measures of goodness of fit, although there is a line of research in this regard, with positive results in recent years (Henseler et al., 2014). Therefore, when there is little knowledge about the relationship of the structural model or the characteristics of the measurement model, or when the study focuses more on exploration than confirmation, PLS-SEM is a better alternative than CB-SEM (Hair et al., 2017).

Future studies are advised to be addressed regarding the following points. The relationship between HRM and IP need more clarification as prior studies contradict this study’s findings leaving unsolved the direction of possible influences between these variables. The role and nature of OC because prior literature and the mediator role it has between HRM and IP raise questions about how it behaves as a composite construct and what exactly are its elements, particularly, in the context of NPHs where the mainstream differs from for-profit organizations and such. Finally, further investigations need to explore the empirical indicators of innovative performance, such as the number of patents registered, the number of new services implemented, or others in order to have a better understanding of this endogenous variable and how it is influenced.

Regarding practical implications, this study reflects an opportunity for NPHs to implement an innovative approach regarding their organizational performance. Technology is often related to innovation that demands organizations the investment of money that sometimes is difficult to count on. This is the case of most non-profit organizations such as non-profit hospitals. For this reason, the resources-based theory (Barney, 1991) pushes companies to take advantage of all their resources to earn the expected performance and develop competitive advantages. This view leads to value the HRM as an element to foster the IP, which could be more affordable in a general basis. Particularly, as human resources influence positively the performance of non-profit organizations without much effort to manage them, a reasonable investment on the staff management might be even more beneficial for an innovative performance.

The findings support the hypotheses remaining some aspects to deepen. HRM is a wide element in the organizational performance that is supposed to manage the OC as well as staff training, compensation, promotion and so forth. Similarly, the organizational climate and principles are related to OC and they are worth being included in the model. Broadening the model considering their dimensions might lead to better understand and predict an innovative performance. Furthermore, assessing the model to other non-profit organizations into the health sector that handle tight budgets as well, for instance nursing homes, might be conclusive about the role of HRM to positive influence IP to enhance quality of life overall.

## Data Availability Statement

The datasets presented in this article are not readily available because data is the property of a third party. Requests to access the datasets should be directed to OL-M, ohlopezm@ut.edu.co.

## Ethics Statement

Ethical review and approval was not required for the study on human participants in accordance with the local legislation and institutional requirements. Written informed consent to participate in this study was provided by the participants.

## Author Contributions

JA-P, OL-M, CS-P, and RZ-T contributed to the conception and design of the study, organized the database, performed the statistical analysis, wrote the first draft of the manuscript, and wrote sections of the manuscript. All authors contributed to the manuscript revision, read and approved the submitted version.

## Conflict of Interest

The authors declare that the research was conducted in the absence of any commercial or financial relationships that could be construed as a potential conflict of interest.
